# Multimodal MRI and ^1^H-MRS for Preoperative Stratification of High-Risk Molecular Subtype in Adult-Type Diffuse Gliomas

**DOI:** 10.3390/diagnostics14222569

**Published:** 2024-11-15

**Authors:** Xin Han, Kai Xiao, Jie Bai, Fengqi Li, Bixiao Cui, Ye Cheng, Huawei Liu, Jie Lu

**Affiliations:** 1 Department of Radiology and Nuclear Medicine, Xuanwu Hospital, Capital Medical University, Beijing 100053, China; rene_oiaty@163.com (X.H.);; 2 Beijing Key Laboratory of Magnetic Resonance Imaging and Brain Informatics, Capital Medical University, Beijing 100053, China; 3 Department of Neurosurgery, Xuanwu Hospital, Capital Medical University, Beijing 100053, China; 4 China Research & Scientific Affairs, GE Healthcare, Beijing 100176, China

**Keywords:** gliomas, isocitrate dehydrogenase, O^6^-methylguanine DNA-methyltransferase, magnetic resonance spectroscopy, diffusion tensor imaging

## Abstract

Isocitrate dehydrogenase (IDH) and O^6^-methylguanine-DNA methyltransferase (MGMT) genes are critical molecular markers in determining treatment options and predicting the prognosis of adult-type diffuse gliomas. **Objectives**: this study aimed to investigate whether multimodal MRI enables the differentiation of genotypes in adult-type diffuse gliomas. **Methods**: a total of 116 adult-type diffuse glioma patients (61 males, 51.5 (37, 62) years old) who underwent multimodal MRI before surgery were retrospectively analysed. Multimodal MRI included conventional MRI, proton magnetic resonance spectroscopy (^1^H-MRS), and diffusion tensor imaging (DTI). Conventional visual features, N-acetyl-aspartate (NAA)/Creatine (Cr), Choline (Cho)/Cr, Cho/NAA, fractional anisotropy (FA), mean diffusivity (MD), and diffusion histogram parameters were extracted on the whole tumour. Multimodal MRI parameters of IDH-mutant and IDH-wildtype gliomas were compared using the Mann–Whitney U test, Student’s *t*-test, or Pearson chi-square tests. Logistic regression was used to select the MRI parameters to predict IDH-mutant gliomas. Furthermore, multimodal MRI parameters were selected to establish models for predicting MGMT methylation in the IDH-wildtype gliomas. The performance of models was evaluated by the receiver operating characteristics curve. **Results**: a total of 56 patients with IDH-mutant gliomas and 60 patients with IDH-wildtype glioblastomas (GBM) (37 with methylated MGMT and 17 with unmethylated MGMT) were diagnosed by 2021 WHO classification criteria. The enhancement degree (OR = 4.298, *p* < 0.001), necrosis/cyst (OR = 5.381, *p* = 0.011), NAA/Cr (OR = 0.497, *p* = 0.037), FA-Skewness (OR = 0.497, *p* = 0.033), MD-Skewness (OR = 1.849, *p* = 0.035), FA_mean_ (OR = 1.924, *p* = 0.049) were independent factors for the multimodal combined prediction model in predicting IDH-mutant gliomas. The combined modal based on conventional MRI, ^1^H-MRS, DTI parameters, and histogram performed best in predicting IDH-wildtype status (AUC = 0.890). However, only NAA/Cr (OR = 0.17, *p* = 0.043) and FA (OR = 0.38, *p* = 0.015) were associated with MGMT methylated in IDH-wildtype GBM. The combination of NAA/Cr and FA-Median is more accurate for predicting MGMT methylation levels than using these elements alone (AUC, 0.847 vs. 0.695/0.684). **Conclusions**: multimodal MRI based on conventional MRI, ^1^H-MRS, and DTI can provide compound imaging markers for stratified individual diagnosis of IDH mutant and MGMT promoter methylation in adult-type diffuse gliomas.

## 1. Introduction

Adult-type diffuse gliomas comprise most primary malignant brain tumours, which include IDH mutants without 1p/19q non-codeleted astrocytoma, IDH mutants with 1p/19q codeleted oligodendroglioma, and IDH-wildtype glioblastoma according to the 2021 WHO classification of central nervous system tumours [1,2]. The IDH mutation is essential for prognostic risk stratification and evaluating treatment response in patients with adult-type diffuse gliomas, correlating with improved overall survival and treatment response relative to IDH-wildtype gliomas [3]. Treatment strategies tailored to distinct molecular subtypes have been shown to enhance patient survival by approximately 17.2 months [4,5]. Furthermore, IDH-wildtype GBM with methylated MGMT has greater sensitivity to temozolomide chemotherapy than those with unmethylated MGMT, which can prolong the survival time of IDH-wildtype GBM by 7.2–8.9 months [6,7]. Histological specimens acquired through resection or biopsy are the current standard method for genotyping and are invasive and time-consuming. Furthermore, the biopsy pathology may be disturbed by intratumor heterogeneity [8,9].

Therefore, a non-invasive and accurate imaging prediction method for glioma genotypes holds excellent potential in clinical practice for patients with adult-type diffuse gliomas. Multimodal MRI, including conventional MRI, DTI, and ^1^H-MRS et al., can non-invasively provide semi-quantitative and quantitative information evaluating the morphology, composition, diffusion, and metabolism characteristics of gliomas. Conventional MRI reflects the size, morphology, and composition of gliomas based on T1-weighted images(T1WI), T2-weighted images(T2WI), fluid-attenuated inversion recovery (FLAIR), and T1WI contrast enhancement(T1CE). Conventional MRI features, including the degree of enhancement, peritumoral oedema, and the presence of cysts, have been shown to predict IDH status in previous studies [10]. Additionally, the proportion of non-enhancing lesions was associated with MGMT methylation in gliomas [11].

^1^H-MRS and DTI are advanced MRI techniques commonly used to diagnose gliomas without needing contrast agents. ^1^H-MRS is designed to provide the ratios of specific metabolites, which consist of NAA/Cr, Cho/Cr, and Cho/NAA. Lower NAA/Cr revealing neuronal dysfunction related to the tumour has been identified as being associated with the grade of gliomas [12,13]. Some studies also found that Cho/Cr and Cho/NAA were positively correlated with Ki-67 levels, indicating glioma cell proliferation [14]. And IDH-wildtype GBM has a higher Ki-67 level than IDH-mutant adult-type diffuse gliomas [15]. Thus, ratios of metabolites based on ^1^H-MRS could supply the potential value for determining IDH and MGMT status in adult-type diffuse gliomas.

In addition, DTI as a technique can assess the restricted diffusion of water molecules in tissues and the degree of anisotropy of water molecule diffusion, thereby reflecting the microscopic pathological characteristics of tumour tissue. Anisotropy parameters include FA, and the MD parameter can reflect the tissue’s average degree of water diffusion [16]. Previous studies have demonstrated the potential of DTI parameters in predicting the WHO grade and IDH status of gliomas [17], and histogram feature analysis can be used to obtain more features that reflect tumour heterogeneity, thereby more comprehensively evaluating the biological characteristics of tumours [18,19].

In recent years, some studies have shown that multimodal MRI has a better prediction performance than mono-modal imaging. Combining MD and Cho/Cr ratios could effectively predict the WHO grade of gliomas (with a sensitivity and specificity of 87.0% and 88.9%, respectively), as described in a previous study [20]. Another study showed that combining FA and MD values and conventional MRI could improve the sensitivity from 59.7% to 92.2% and the specificity from 52.9% to 75.8% of the IDH mutation prediction model [21]. However, studies have yet to explore the value of combining conventional MRI visual features and DTI and ^1^H-MRS quantitative parameters for the prediction of molecular typing of gliomas, especially for the genotype group of adult-type diffuse gliomas based on the fifth edition of the WHO classification.

Thus, this study aimed to investigate whether multimodal MRI analysis enables differentiation of IDH mutation in patients with adult-type diffuse gliomas and methylation status of MGMT promoters in GBM patients and to establish non-invasive MRI imaging markers for preoperative stratification of high-risk molecular subtypes in adult-type diffuse gliomas.

## 2. Materials and Methods

### 2.1. Study Patients

**Figure 1:**
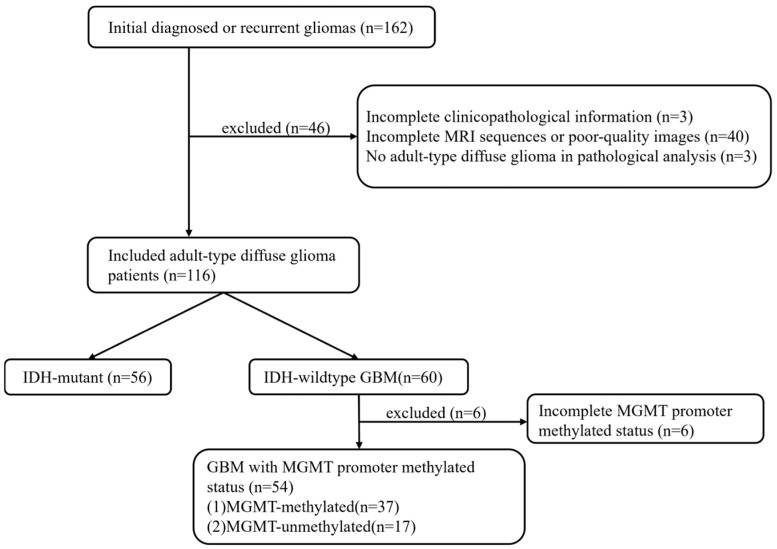
Flow chart of the inclusion and exclusion criteria.

Between February 2019 and June 2024, 162 adult patients who underwent an MRI brain scan within one week before biopsy or surgical resection with initial or recurrent gliomas, confirmed by pathological examination of our institution, were included in this retrospective analysis study. Figure 1 shows the patient enrolment process. There were 46 cases excluded because of incomplete clinicopathological information (n = 3), incomplete MRI sequences, or poor-quality images (*n* = 40), or no adult-type diffuse glioma in the pathology (*n* = 3). A final total of 116 patients with adult-type gliomas (mean age 51.5, range 19–89 years; 61 males, 55 females) were enrolled for this study. According to the IDH genotypes and the MGMT promoter status, patients were initially categorised into the IDH-wildtype group (*n* = 60) and the IDH-mutant group (*n* = 56). Then, the patients in the IDH-wildtype group (*n* = 54, due to 6 patients whose MGMT methylation status information was missing) were divided into the MGMT-methylated subgroup (*n* = 37) and the MGMT-unmethylated subgroup (*n* = 17). For this retrospective investigation, the Xuanwu Hospital Medical Ethics Committee authorised the subjects’ informed consent exemption ([2023]044).

### 2.2. Imaging Acquisition

All participants underwent preoperative MR scans, which included conventional MRI sequences, DTI, and ^1^H-MRS using a hybrid time-of-flight (TOF) PET/MR (GE Signa 750w, GE Healthcare, Milwaukee, WI, USA) scanner equipped with a 19-channel head–neck coil. The conventional MRI scans comprised axial T1-weighted (T1WI), axial T2-weighted (T2WI), T2-weighted fluid-attenuated inversion recovery (T2 FLAIR), axial diffusion-weighted imaging (DWI), 3D T1, and a 3D contrast-enhanced T1-weighted sequence (3D T1CE). Detailed MRI scan parameters are provided in Supplementary Table S1.

The DTI sequence parameters were as follows: TR/TE = 9232/97 ms, FOV = 224 mm × 224 mm, layer thickness = 3.5 mm, matrix = 112 × 112, b value = 0 and 1000 s/mm^2^ with diffusion encoding in 64 directions for every b value, resulting in a total scanning time of 11 min and 32 s.

For ^1^H-MRS, we utilised the vendor-provided point-resolved spectroscopy (PRESS) sequence a for two-dimensional magnetic resonance spectroscopic imaging (2D MRSI). A 2 cm thick slice was positioned axially at the location where the solid portion of the tumour appeared largest. The volume of interest (VOl) was selected by a combination of the PRESS sequence (which involves the slice-selective signal excitation techniques) and outer volume suppression using 6 outer volume saturation bands. The acquisition parameters were as follows: TR/TE = 1000/144 ms, FOV = 240 mm × 240 mm, matrix size = 18 × 18, slice thickness = 20.0 mm, and a number of excitations (NEX) = 1. The voxel size was approximately 1.3 cm × 1.3 cm × 2.0 cm, consistent across both acquisition and reconstruction. The ^1^H-MRS scan time was approximately 5 min and 28 s. T2 FLAIR images were used for precise localization of MRS. Signal intensities were calculated by the sum of each peak with the metabolite peaks assigned to the following chemical shift ranges: Cr at 3.12–2.97 ppm, Cho at 3.34–3.19 ppm and NAA at 2.13–1.96 ppm.

### 2.3. Imaging Analysis

The conventional MRI features included tumour location, enhancement degree (none, mild, marked), presence of necrosis/cyst change, presence of nonenhancing tumour and presence of peritumoral oedema, and the peritumoral oedema was graded based on the maximum distance between the tumour margin and oedema: 0 for not apparent (≤1 cm), 1 for mild to moderate (>1 cm and ≤2 cm), and 2 for severe (>2 cm) [22]. Two radiologists, blinded to the patient’s molecular and clinical data, reviewed the MR images and assessed the tumour’s radiologic features by consensus. Necrosis was defined as an area within the tumour’s CE region with little or no contrast enhancement. The cystic regions were homogeneous regions that were isointense to the cerebrospinal fluid on T1WI and T2WI images and had an enhancing rim on T1 CE images [23,24].

The GE AW4.7 workstation analysed the ^1^H-MRS data with an auto-chemical shift protocol. The workstation automatically generated the NAA/Cr, Cho/Cr and Cho/NAA ratio. The region of interest (ROI) was placed on the area showing the solid portion of the tumour on the conventional MRI sequence. The cystic or necrotic portion and haemorrhagic regions were carefully avoided, and we selected one to three ROIs to calculate the averaged metabolite ratios [14].

**Figure 2:**
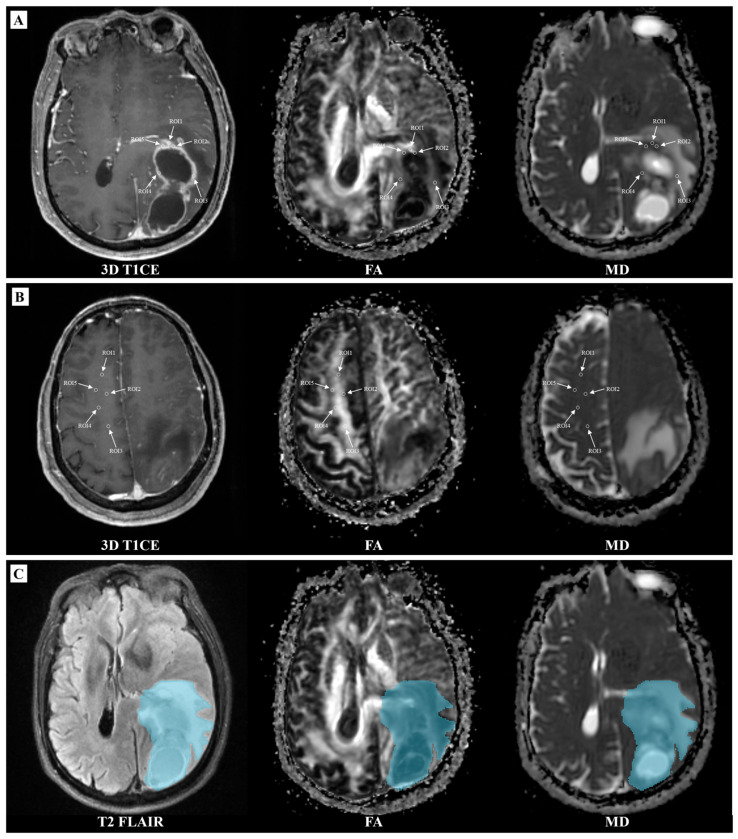
Delineation of tumour regions of interest and volume of interest; (**A**) Delineation of the ROI within the solid part of a glioma; (**B**) Delineation of the ROI within the contralateral frontal part of the centrum semiovale; (**C**) Delineation of the VOI for the whole tumor area. ROI, region of interest; VOI, volume of interest; three-dimensional T1CE, three-dimensional contrast-enhanced T1-weighted sequence; T2 FLAIR, T2-weighted fluid-attenuated inversion recovery; FA, fractional anisotropy; MD, mean diffusivity.

The GE AW 4.7 workstation calculated FA and MD maps based on DTI. Then, FLAIR images, FA and MD maps were registered to T1CE images for subsequent analyses using SlicerANTs extension in 3D slicer (v5.4.0, https://www.slicer.org accessed on 28 September 2024). Five ROIs were manually delineated on the solid part of each lesion, avoiding overlap and excluding necrotic, haemorrhagic and cystic zones, to calculate FA and MD maps and the tumour-to-normal tissue values ratio. Each ROI was sized between 20 and 40 mm^2^, with an additional five ROIs placed on the contralateral frontal part of the centrum semiovale, consistent with previous studies [25]. For FA and MD metrics, we calculated the average value for minimum, maximum, mean, and median value in each ROI, representing them as FA_min_, FA_mean_, FA_median_ and FA_max_ (for Fractional Anisotropy), MD_min_, MD_mean_, MD_median_ and MD_max_, (for Mean Diffusivity). The relative FA (rFA) and relative MD (rMD) for minimum, mean, median and maximum were calculated by dividing the tumour FA and MD value by those of the centrum semiovale, as shown in Figure 2A,B.

Histogram features of FA and MD maps were extracted based on the VOI that was manually delineated around the whole tumour region, including peritumoral oedema by two radiologists (with eight years of experience in neuro-oncology) using the SlicerRadiomics in 3D slicer (v5.4.0, https://www.slicer.org accessed on 28 September 2024), as shown in Figure 2C. The histogram features included 10thPercentile, 90thPercentile, Minimum, Maximum, Mean, Median, Range, Interquartile Range, Energy, Total Energy, Entropy, Kurtosis, Skewness, Mean Absolute Deviation, Robust Mean Absolute Deviation, Root Mean Squared, Uniformity, and Variance.

### 2.4. Pathological Histological and Molecular Analysis

Histopathological and molecular results were retrospectively confirmed by one pathologist with more than ten years of experience in neuro-oncology. The classification and grading of the included gliomas were reconfirmed according to the 2021 WHO classification. The IDH mutation status of gliomas was determined by immunohistochemistry or pyrophosphate sequencing. Fluorescence quantitative PCR was applied to detect the status of MGMT promoter methylation.

### 2.5. Models Establishment and Statistical Analyses

All continuous quantitative data were tested for normality using Kolmogorov-Smirnov’s test. Non-normally distributed variables were represented by the median (interquartile range), while normally distributed variables were represented by mean ± standard deviation. The Mann–Whitney U/Student’s *t*-test or Pearson chi-square/Fisher’s exact test was used to initially select conventional MRI, ^1^H-MRS, DTI conventional parameters, and DTI histogram features correlated with the molecular type. To reduce the dimensions of the retained features, univariate logistic regression analysis and least absolute shrinkage selection operator (LASSO) regression were used for developing the conventional MRI model, MRS model, conventional DTI model, and DTI histogram model. Finally, the logistic regression combined with 5-fold cross-validation was used to fit the prediction models of each MRI and the combined prediction model, which integrated predictors from each model. Receiver operating characteristic (ROC) curve analysis was performed to evaluate the models, with performance metrics (AUC, sensitivity, specificity, accuracy) reported as the average from 5-fold cross-validation. The SPSS software (version 28.0, SPSS, Inc., Chicago, IL, USA) and R software (version 4.3.3, R Foundation for Statistical Computing, Vienna, Austria) were used collaboratively to perform feature selection, model establishment, statistical analysis, and model evaluation. A *p* value of less than 0.05 was considered statistically significant in all statistical tests.

## 3. Results

### 3.1. Clinicopathological Characteristics

The characteristics of the included patients are summarised in Table 1. Out of 116 patients, 35 (30.17%) had WHO grade 2 gliomas, 11 (9.48%) had WHO grade 3 gliomas, and 70 (60.34%) patients had GBM. Of all 116 patients, 56 were diagnosed with IDH-mutant gliomas and 60 with IDH-wildtype GBM. For patients diagnosed with GBM, 37 were diagnosed with methylated MGMT and 17 with unmethylated MGMT.

### 3.2. Models Development for Predicting IDH

**Figure 3:**
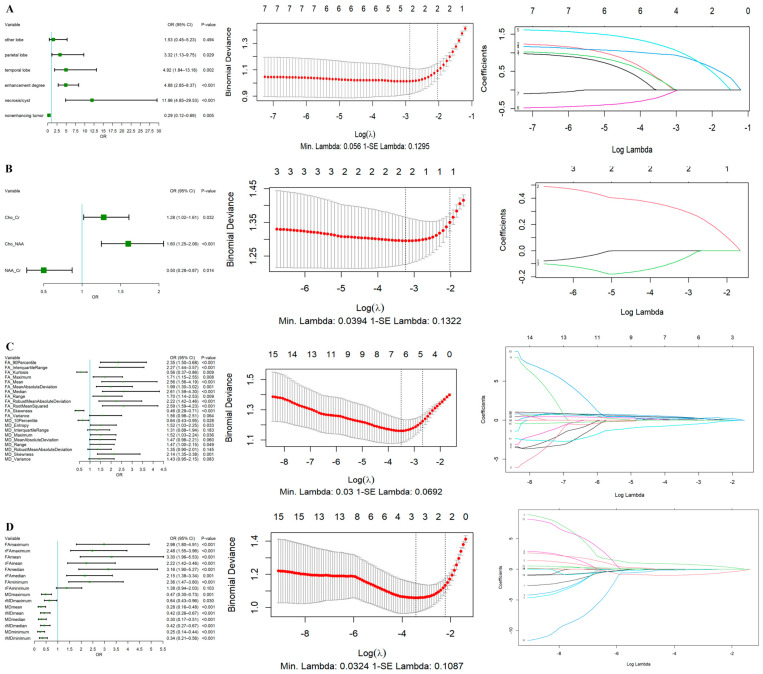
Forest plot of univariate logistic regression and the results of LASSO regression for different modalities MRI. (**A**) Conventional MRI results. (**B**) ^1^H-MRS results. (**C**) DIT histogram results. (**D**) Conventional DTI results.

To compare the predictive performances of the different modalities’ models, logistic regression models were constructed, respectively, using features selected by the MU test, univariate logistic regression, and LASSO selection from the conventional MRI, MRS, DTI histogram, and conventional DTI. In contrast, the combined prediction model was constructed using their combined features. Figure 3 shows the results of univariate logistic regression and the LASSO regression for different modalities of MRI. The detailed feature selection data and logistic regression results for mono-modal MRI were provided in Supplementary Tables S2 and S3.

After using logistic regression to acquire the best combination of features for combined model, enhancement degree (OR = 4.298, *p* < 0.001) and necrosis/cyst (OR = 5.381, *p* = 0.011) from the conventional MRI, NAA/Cr(OR = 0.497, *p* = 0.037) from the MRS, FA-Skewness (OR = 0.497, *p* = 0.033) and MD-Skewness (OR = 1.849, *p* = 0.035) from the DTI histogram features and FA_mean_ (OR = 1.924, *p* = 0.049) from conventional DTI were selected as independent risk factors for predicting IDH-wildtype status in the combined multimodal model (Table 2).

**Figure 4:**
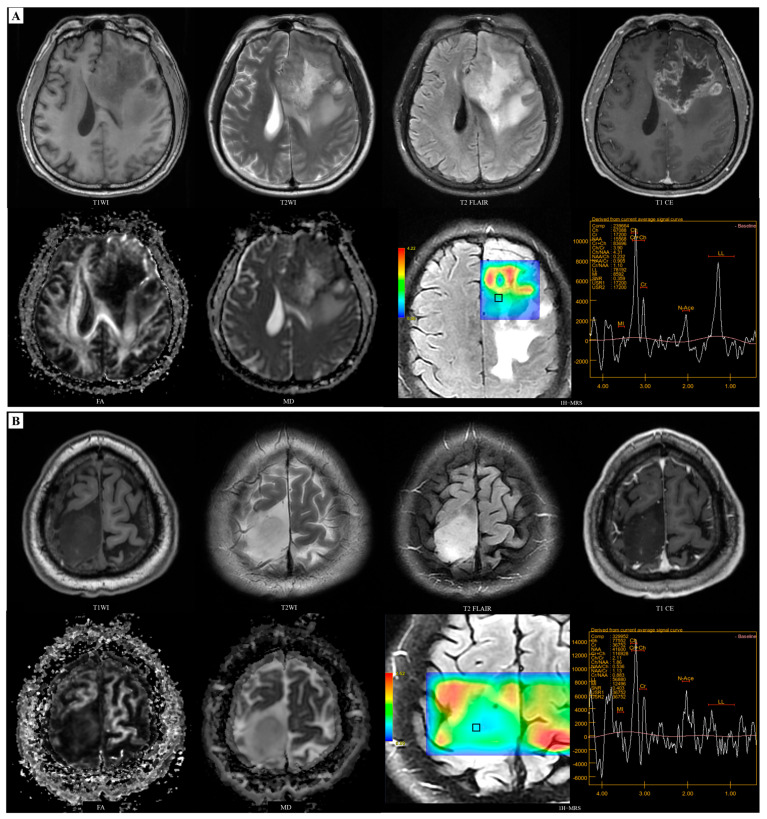
Multimodal MRI of IDH-wildtype and IDH-mutant adult-type diffuse glioma cases. (**A**) A 59-year-old male patient, IDH-wildtype GBM: the mass in the left frontal lobe is isointense or hypointense on T1WI and heterogeneously hyperintense on T2WI. The tumour parenchyma show marked circular enhancement on the enhanced scan, with severe oedema and necrosis, with relatively high FA_mean_ (0.163) and low MD_min_ (558.04 × 10^−6^ mm^2^/s). ^1^H-MRS analysis (voxel size = 1.3 cm × 1.3 cm × 2.0 cm) showed relatively low level of NAA/Cr (0.905). (**B**) A 38-year-old male patient, IDH-mutant adult-type diffuse glioma: the mass located in the right frontal lobe with circular hypointense on T1WI and hyperintense on T2WI, and no enhancement on enhanced scans, with the absence of peritumoral oedema and necrosis, with relatively low FA_mean_ (0.101) and high MD_min_ (1386.08 × 10^−6^ mm^2^/s). ^1^H-MRS analysis (voxel size = 1.3 cm × 1.3 cm × 2.0 cm) showed moderate level of NAA/Cr (1.13).

The comparison of selected features of different IDH genotypes of patients with adult-type diffuse gliomas is shown in Table 3. Marked enhancement was significantly more prevalent in the IDH-wildtype group (76.6% vs. 25%, *p* < 0.001), as was necrosis/cyst formation (85% vs. 32.1%, *p* < 0.001). Additionally, the NAA/Cr ratio was significantly lower in the IDH-wildtype group compared to the IDH-mutant group (0.93 vs. 1.36, *p* < 0.001). Regarding DTI histogram features, the IDH-mutant group exhibited significantly higher FA-Skewness than the IDH-wildtype group (1.38 vs. 0.92, *p* < 0.001). Meanwhile, the IDH-wildtype group showed significantly elevated MD-Skewness compared to the IDH-mutant group (0.725 vs. 0.451, *p* = 0.001). We observed a considerably lower FA_mean_ value in the IDH-mutant group than in the IDH-wildtype group (0.10 vs. 0.18, *p* < 0.001). Typical cases of adult-type diffuse glioma patients are depicted in Figure 4 and see Supplementary Figure S1 for the ROI selection in the multi-voxel 2D MRSI spectra of cases.

### 3.3. Models Development for Predicting MGMT

**Figure 5:**
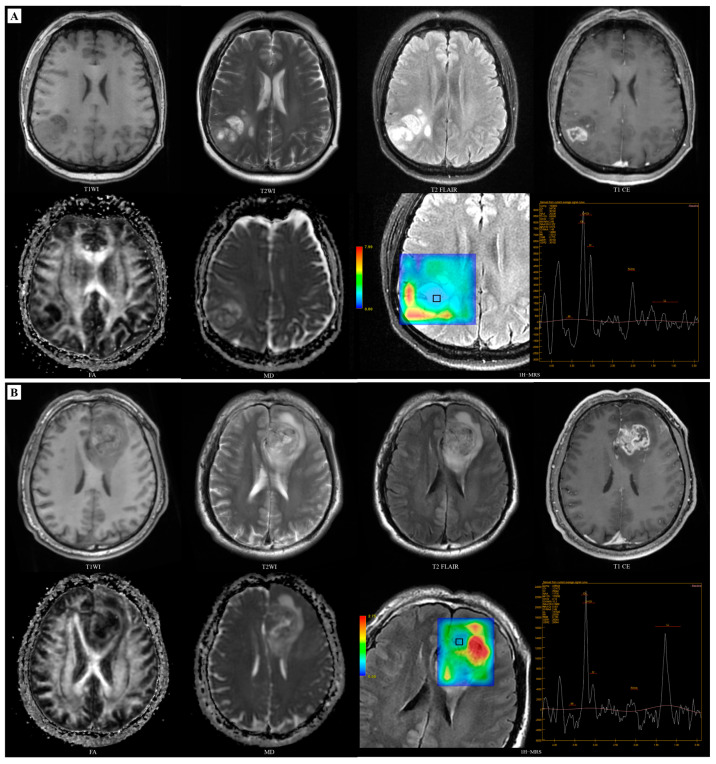
Multimodal MRI of MGMT-methylated and MGMT-unmethylated IDH-wildtype GBM cases. (**A**) A 54-year-old female patient, IDH-wildtype with MGMT methylated: the mass located in the right parietal lobe with long T1 and long T2 signals. The tumour parenchyma show obvious-heterogeneous enhancement, with peritumoral oedema and necrosis, with FA-Median = 0.215. ^1^H-MRS analysis (voxel size = 1.3 cm × 1.3 cm × 2.0 cm) showed the level of NAA/Cr is 0.674. (**B**) A 50-year-old male patient, IDH-wildtype with MGMT unmethylated: the mass located in the left frontal lobe with circular long T1 and long T2 signals and obvious heterogeneous enhancement on enhanced scans like the previous case, with the presence of peritumoral oedema and necrosis, with FA-Median = 0.176. ^1^H-MRS analysis (voxel size = 1.3 cm × 1.3 cm × 2.0 cm) showed the level of NAA/Cr is 0.401.

For MGMT promoter methylation status prediction, only NAA/Cr from the ^1^H-MRS quantitative analysis and FA-Median from the DTI histogram features were retained and then used as predictors. NAA/Cr (1.03 vs. 0.72, *p* = 0.014) and FA-Median (0.172 vs. 0.162, *p* = 0.026) were higher and significantly different in the MGMT-methylated group than in the MGMT-unmethylated group. These features were respectively entered into the prediction model. Table 4 shows the univariate and combined multivariate logistic regression results for MGMT promoter methylation status predictors. Figure 5 shows cases of glioblastoma patients with different methylation status of the MGMT promoter and refer to Supplementary Figure S2 for the ROI selection in the multi-voxel 2D MRSI spectra of the cases. The detailed feature selection data were provided in Supplementary Table S4.

### 3.4. Performance of Prediction Models

**Figure 6:**
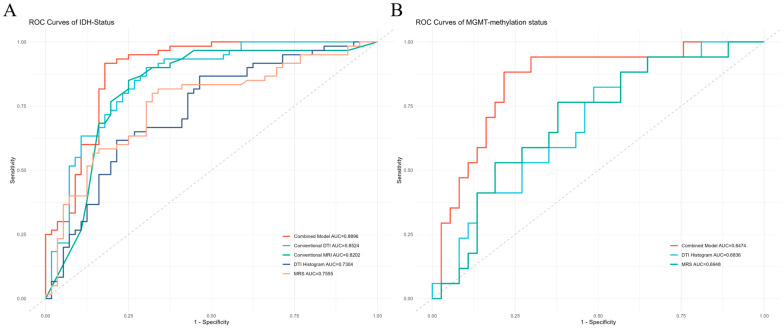
ROC curves for diagnostic performance of prediction models. (**A**) ROC curve for IDH status prediction in the 5-fold cross-validation. (**B**) ROC curve for MGMT status prediction in the 5-fold cross-validation. ROC is the receiver operating characteristic. AUC is the area under the curve.

IDH genotypes prediction. To assess the diagnostic performance of each prediction model, we calculated AUC, sensitivity, specificity, and accuracy in ROC analysis using the approach of 5-fold cross-validation (Table 5). The combined model showed the highest AUC value (0.890) among these models. The best diagnostic performance of a mono-modal MRI prediction model was conventional DTI parameters (AUC = 0.852), followed by the conventional MRI model (AUC = 0.820), ^1^H-MRS model (AUC = 0.756), and the DTI histogram model (AUC = 0.730), as shown in Figure 6A.

MGMT promoter methylation status prediction. We also calculated AUC, sensitivity, specificity, and accuracy via 5-fold cross-validation to assess the diagnostic performance of each monomodal MRI and the combined prediction model (Table 5). A mono-modal MRI prediction model’s most excellent diagnostic performance was the ^1^H-MRS model (AUC = 0.695), followed by the DTI histogram model (AUC = 0.684). The combined prediction model had superior performances to the two kinds of monomodal MRI prediction model (AUC = 0.847), as shown in Figure 6B.

## 4. Discussion

The 2021 WHO classification of central nervous system tumours defined adult-type diffuse gliomas based on molecular information. So, investigating an effective and non-invasive approach for predicting glioma genotyping is crucial for the personalised treatment and prognosis assessment of glioma patients. We used machine learning methods combining conventional and advanced MRI techniques to establish the stratified diagnosis system of high-risk genotypes in adult-type diffuse gliomas, which is consistent with current clinical diagnosis and practical treatment strategies. Our findings indicated that the logistic regression model of conventional MRI visual features, metabolite ratios from ^1^H-MRS quantitative analysis, histogram features of DTI, and conventional parameters of DTI could be used to predict IDH genotypes individually, and we also found that the combined model performed better than monomodal MRI prediction model. The combined model showed acceptable results regarding MGMT promoter methylation status prediction. Still, the NAA/Cr and FA-Median could not individually predict the MGMT promoter methylation status well, according to the present study.

On conventional MRI, our study revealed that IDH-wildtype gliomas were more likely to show marked enhancement and have necrotic or cystic components compared to IDH-mutant gliomas; this is consistent with radiologic features of gliomas reported in the previous literature [26,27]. In IDH-wildtype gliomas, this appearance may be associated with inhomogeneous growth and the upregulation of hypoxia-inducible factor 1 subunit alpha and vascular endothelial growth factor, which were associated with angiogenesis within contrast-enhancement regions [28]. Previous studies have revealed the predictive value of conventional MRI in the IDH genotype; in the study by Zhou et al. [26], they constructed a random forest model with the proportion of necrosis and lesion size, which was used as the optimal set of features included, which reached an AUC of 0.73. Moreover, Xiong et al. [21] proved that a combination of conventional MR and DTI can improve genotyping accuracy for IDH status. By combining the visual features of tumour location and enhancement with the FA and MD values, they found that the sensitivity and specificity of the prediction model, respectively, increased from 59.7% to 52.9% and from 92.2% to 75.8% compared to conventional MRI features alone. In this study, two features were selected from the DTI histogram and one from conventional DTI parameters for predictors in the prediction model. DTI measures the degree of anisotropic diffusion of water molecules within the glioma, thereby indirectly reflecting the density and heterogeneity of the tumour cells and providing quantitative information on abnormal water molecule diffusion for predicting IDH mutations in gliomas [29]. Previous studies have shown that compared to the IDH-wildtype group, the FA value of the IDH-mutant group is considerably lower than in the IDH-wildtype group. This finding is consistent with the results obtained in the present study [21] and this may be because the greater density of tumour cells and higher levels of tumour angiogenesis in IDH-wildtype gliomas leads to a greater degree of anisotropy and restricted diffusion of water molecules [30]. Later, other studies employed the histogram features of parameters of DTI in the whole tumour region to predict the IDH status of gliomas and demonstrated the feasibility of this approach. In a small sample study, Huang et al. [30] constructed a model based on histogram features of FA and MD in the whole region of the tumour. They found that histogram features such as the mean, 25th percentile, 75th percentile, and skewness can predict IDH status. Among the single-parameter features, the 25th percentile of FA has the highest AUC of 0.87. After combining the parameters, the AUC of the model reached 0.93. Despite the higher diagnostic efficacy of the model than our DTI histogram prediction model, this study only included 40 patients. It was limited to insular lobe gliomas, so the predictive performance of the IDH genotype of gliomas in other brain regions was not explored. Additionally, Gao et al. [17] discovered that the histogram of DTI parameters could accurately predict the IDH genotype of gliomas in their prospective study (AUC = 0.76). Skewness measures the asymmetry of the distribution of values about the mean value. In our research, the FA-Skewness of the IDH-mutant gliomas is significantly higher than that of the IDH-wildtype group. In contrast, compared to the IDH-mutant group, the MD-Skewness of the IDH-wildtype group is considerably higher, indicating that the FA histogram distribution of IDH-mutant gliomas and the MD histogram distribution of IDH-wildtype gliomas is more skewed, as Huang et al. reported previously [31]. The higher MD-Skewness is associated with higher-grade gliomas in earlier studies, which may be caused by the higher capillary network density and tumour cellularity density [32,33].

In contrast to DTI, which assesses variations in the degree and direction of water molecule diffusion due to tumour cell proliferation or angiogenesis, ^1^H-MRS provides a direct assessment of tissue differences among tumours of different molecular genotypes by quantifying metabolite levels. Few studies predicted IDH mutations by common metabolite ratios in the ^1^H-MRS. A pilot study including 24 cases showed that the NAA/Cr could predict IDH-mutant status (AUC = 0.808) [34]. Several studies have indicated that ^1^H-MRS was capable of accurately predicting the IDH genotype of gliomas by measuring the relative concentration of 2-hydroxyglutarate (2-HG), which is a kind of oncometabolite with high levels of IDH-mutant gliomas [35,36]. However, determining the 2-HG peak is technically challenging, and phantom experiments were required to ensure that 2-HG could be accurately detected and distinguished from other metabolites [37]. Furthermore, one study has indicated that the sensitivity of ^1^H-MRS for detecting 2-HG was influenced by tumour volume [38]. Therefore, our study combined conventional MRI and DTI quantitative parameters with ^1^H-MRS to predict IDH status better. This study demonstrated that the AUC of this mono-modal ^1^H-MRS prediction model was 0.756 with a sensitivity and specificity of 76.7%, which is lower than that reported in a meta-analysis representing the diagnostic potential of 2-HG ^1^H-MRS with the pooled sensitivity of 93% and the specificity of 84%. However, after combining it with conventional MRI features and DTI quantitative parameter features, the sensitivity and specificity of the combined model increased to 93.3% and 84.1%, respectively, which shows that the application of multimodal MRI combined models can compensate for the shortcomings of ^1^H-MRS. Therefore, it enhances the overall diagnostic accuracy.

Methylation of the MGMT promoter can increase the killing effect of the alkylating agent chemotherapy drugs on tumour cells, reduce tumour cell proliferation and repair to prolong patients’ survival [39]. In particular, the GBM with MGMT promoter methylation is more sensitive to chemotherapeutic drugs such as temozolomide, which can prolong overall survival by approximately 6.4 months [40]. Therefore, the non-invasive assessment of MGMT methylation in gliomas is essential to guide the selection of individualised chemotherapeutic agents. Our research has shown no significant difference in conventional MRI features between MGMT-methylated and MGMT-unmethylated glioblastomas, which matches the previously reported findings [22,24,41]. Latysheva et al. [40] found no significant difference between the MGMT methylation group and the MGMT-unmethylated group in FA and MD parameters in enhancement regions of the tumour by analysing DTI quantitative parameters in 42 cases of GBM, which is also aligns with our results. However, in the present study, we found between-group differences in the FA-Median histogram feature of whole-tumour regions. This result was inconsistent with the results of previous studies [30], and different cohorts may partially account for these controversial findings. The value of ^1^H-MRS in predicting the methylation status of the MGMT promoter in GBM has not been reported to our knowledge. In a previous study, it was found [42] that NAA/Cr in the postoperative peritumoral oedema zone of GBM could serve as a predictor (OR = 0.379, *p* < 0.001). The present study found a significant difference in NAA/Cr between the MGMT-methylated and the MGMT-unmethylated groups. Interestingly, after including NAA/Cr and FA-Median as predictors in our study, the performance of the combined prediction model was acceptable (AUC = 0.847). A previous meta-analysis by Doniselli reported that radiomic models were not robust enough to accurately predict MGMT promoter methylation status in glioma patients before surgery with a pooled AUC of 0.78 [43]. Our results may provide a new biomarker to predict MGMT promoter status in patients with GBM.

In addition to the retrospective design, there are some other limitations. Firstly, our study includes a relatively low number of patients, especially in GBM patients with complete MGMT promoter status. Although we did not have a validation cohort, we validated the stability of prediction models using a 5-fold cross-validation method. We will continue to collect relevant samples and overcome this limitation by conducting multicentre research. Secondly, before extracting histogram features in our study, we manually performed the segmentation of the VOI, which can introduce a certain level of subjectivity and influence results. In the future, deep learning algorithms could be used to segment tumour lesions automatically. Finally, the prognosis for overall or progression-free survival was not evaluated due to insufficient prognostic data, which needs further exploration in future studies.

## 5. Conclusions

The DTI and ^1^H-MRS parameters show more powerful evaluation potential for IDH mutation and MGMT promoter methylation than conventional MRI. Moreover, the combination of conventional and advanced MRI provides essential value for the stratified diagnosis of high-risk molecular typing in adult-type diffuse gliomas. Nevertheless, further prospective studies are necessary to validate these findings and establish their correlation with clinical outcomes.

## Figures and Tables

**Table 1:** Baseline clinical information of the included patients.

Characteristic	*N* = 116
Sex, *n* (%)	
female	55 (47.41%)
male	61 (52.59%)
Age, years (median [IQR])	51.5 (37, 62)
WHO Grade, *n* (%)	
2	35 (30.17%)
3	11 (9.48%)
4	70 (60.34%)
IDH-status, *n* (%)	
mutant	56( 48.28%)
wildtype	60 (51.72%)
GBM-MGMT, *n* (%)	
methylated	37 (68.52%)
unmethylated	17 (31.48%)

Note: IQR, interquartile range; IDH, isocitrate dehydrogenase; GBM, glioblastoma; MGMT, O^6^-methylguanine-DNA methyltransferase.

**Table 2:** Logistic regression analysis of multimodal MRI for predicting IDH genotypes.

Variable		OR with CI	SE	Wald	*p* Value
**Conventional MRI**	enhancement degree	4.298 (1.962–9.412)	0.4	13.287	<0.001
	necrosis/cyst	5.381 (1.476–19.622)	0.66	6.5	0.011
**^1^** **H-MRS**	NAA/Cr	0.497 (0.258–0.957)	0.334	4.369	0.037
**DTI histogram**	FA-Skewness	0.497 (0.261–0.946)	0.329	4.527	0.033
	MD-Skewness	1.849 (1.046–3.27)	0.291	4.469	0.035
**Conventional DTI**	FA_mean_	1.924 (1.002–3.695)	0.753	3.861	0.049

Note: IDH, isocitrate dehydrogenase; NAA, N-acetyl-aspartate; Cr, creatine; FA, fractional anisotropy; MD, mean diffusivity.

**Table 3:** The multimodal predictors in IDH genotypes of adult-type diffuse gliomas.

Variable		IDH-Mutant *N* = 56	IDH-Wildtype *N* = 60	*Z/t/χ^2^*	*p* Value
**Conventional MRI**	**enhancement degree, *n*** (**%**)			43.58	<0.001
	marked	14 (25.0%)	46 (76.6%)		
	mild	6 (10.7%)	10 (16.7%)		
	no	36 (64.2%)	4 (6.67%)		
	**necrosis or cyst, *n*** (**%**)			33.58	<0.001
	no	38 (67.8%)	9 (15.0%)		
	yes	18 (32.1%)	51 (85.0%)		
**^1^** **H-MRS**	NAA/Cr (median [IQR])	1.36 (1.058, 1.75)	0.93 (0.576, 1.195)	−4.818	<0.001
**DTI histogram**	FA-Skewness (median [IQR])	1.38 (0.96, 1.86)	0.92 (0.69, 1.31)	−3.90	<0.001
	MD-Skewness (median [IQR])	0.451 (−0.202, 0.662)	0.725 (0.308, 1.249)	−3.193	0.001
**Conventional DTI**	FA_mean_ (median [IQR])	0.10 (0.08, 0.13)	0.18 (0.14, 0.22)	−5.39	<0.001

Note: IDH, isocitrate dehydrogenase; IQR, interquartile range; NAA, N-acetyl-aspartate; Cr, creatine; FA, fractional anisotropy; MD, mean diffusivity.

**Table 4:** Logistic regression for predicting MGMT promoter methylation in GBM patients.

Variable	Univariate		Multivariate	
	OR with CI	*p* Value	OR with CI	*p* Value
NAA/Cr	0.17 (0.03–0.95)	0.043	0.10 (0.02–0.56)	0.009
FA-Median	0.38 (0.17–0.83)	0.015	0.22 (0.08–0.64)	0.006

Note: MGMT, O^6^-methylguanine-DNA methyltransferase; NAA, N-acetyl-aspartate; Cr, creatine; FA, fractional anisotropy.

**Table 5:** Performance of models for predicting IDH mutation or MGMT promoter methylation in the 5-fold cross-validation.

Models	IDH-Status				MGMT-Methylation Status			
	AUC	SEN	SPE	ACC	AUC	SEN	SPE	ACC
Conventional MRI	0.820	83.3%	80.5%	81.9%	-	-	-	-
^1^H-MRS	0.756	76.7%	76.7%	76.7%	0.695	90.5%	65.0%	74.4%
DTI histogram	0.730	71.7%	78.2%	74.9%	0.684	90.0%	65.4%	72.2%
Conventional DTI	0.852	92.7%	78.4%	85.6%	-	-	-	-
Combined model	0.890	93.3%	84.1%	88.9%	0.847	88.3%	86.4%	86.9%

Note: IDH, isocitrate dehydrogenase; MGMT, O^6^-methylguanine-DNA methyltransferase; AUC, the area under the curve; SEN, sensitivity; SPE, specificity; ACC, accuracy.

## Data Availability

The datasets used and/or analysed during the current study are available from the corresponding author on reasonable request.

## Supplementary Materials

The following supporting information can be downloaded at https://www.mdpi.com/article/10.3390/diagnostics14222569/s1, Table S1: Conventional MRI scan parameters; Table S2: Features differences from mono-modal MRI between IDH-mutant group and IDH-wildtype group; Table S3: Logistic regression results for prediction IDH status based on features from mono-modal MRI model; Table S4: Features differences from mono-modal MRI between MGMT-methylated group and MGMT-unmethylated group; Figure S1: The ROI selection in the multi-voxel 2D MRSI spectra for cases in the IDH-wildtype group and IDH-mutant group; Figure S2: The ROI selection in the multi-voxel 2D MRSI spectra for cases in the MGMT-methylated group and MGMT-unmethylated group.
