# Supplementary material for: Multimodal MRI and 1H-MRS for Preoperative Stratification of High-Risk Molecular Subtype in Adult-Type Diffuse Gliomas
Source: Diagnostics (Basel). 2024 Nov 15;14(22):2569. doi: 10.3390/diagnostics14222569 (PMC11592885; doi:10.3390/diagnostics14222569)
Supplement: Supplementary file 1 [file diagnostics-14-02569-s001.zip › diagnostics-3258470-supplementary.pdf]

Supplementary Table S1. Conventional MRI scan parameters.

| MR sequences | TE (ms) | TR (ms) | Thickness/Space (mm) | FOV (cm) | Matrix | Nex | Slice |
|---|---|---|---|---|---|---|---|
| Ax T2WI | Minimum | 5000 | 5.0/1.0 | 24×24 | 288×288 | 1 | 22 |
| Ax T2 FLAIR | 140 | 6200 | 5.0/1.0 | 24×24 | 256×256 | 1 | 22 |
| Ax T1WI | 24 | 3002.7 | 5.0/1.0 | 24×24 | 288×256 | 1 | 22 |
| Ax DWI b=1000 | Minimum | 5000 | 5.0/1.0 | 24×24 | 128×128 | 2 | 44 |
| 3D T1 GRE | Minimum | 2100 | 1.0/0.0 | 25×25 | 256×256 | 1 | 188 |
| 3D T1 CE GRE | Minimum | 2100 | 1.0/0.0 | 25×25 | 256×256 | 1 | 188 |

Note: TE, Echo Time; TR, Repetition Time; FOV, Field of View; NEX, Number of Excitations; FSE, Fast Spin Echo; GRE, Gradient Echo; FLAIR: Fluid Attenuated Inversion Recovery; DWI, Diffusion-Weighted Imaging.

Supplementary Table S2. Features differences from mono-modal MRI between IDH-mutant group and IDH-wildtype group.

| Variable | IDH-mutant<br>N = 56 | IDH-wildtype<br>N = 60 | Z/t | *p* value |
|---|---|---|---|---|
| **Conventional MRI** | | | | |
| Sex, n (%) | | | 0.028 | 0.868 |
| female | 27 (48.2%) | 28 (46.7%) | | |
| male | 29 (51.8%) | 32 (53.3%) | | |
| Age, Median (IQR) | 44.5 (34, 54) | 58.5 (48, 65) | -13.149 | <0.001 |
| Tumor location, n (%) | | | 12.532 | 0.006 |
| Frontal lobe | 34 (60.7%) | 19 (31.7%) | | |
| Parietal lobe | 7 (12.5%) | 13 (21.7%) | | |
| Temporal lobe | 8 (14.3%) | 22 (36.7%) | | |
| Other | 7(12.5%) | 6 (10%) | | |
| Enhancement degree, n (%) | | | 43.58 | <0.001 |
| marked | 14 (25%) | 46 (76.6%) | | |
| mild | 6 (10.7%) | 10 (16.7%) | | |
| no | 36 (64.2%) | 4 (6.67%) | | |
| Necrosis or cyst, n (%) | | | 33.578 | <0.001 |
| no | 38 (67.8%) | 9 (15%) | | |
| yes | 18 (32.1%) | 51 (85%) | | |
| Nonenhancing part, n (%) | | | 8.148 | 0.004 |
| no | 9 (16.1%) | 24 (40%) | | |
| yes | 47 (83.9%) | 36 (60%) | | |
| Peritumoral edema, n (%) | | | 28.75 | <0.001 |
| 0 | 34(60.7%) | 8(13.3%) | | |
| 1 | 15(26.8%) | 30(50%) | | |

| | | | | |
|---|---|---|---|---|
| 2 | 7(12.5%) | 22(36.7%) | | |
| **MRS metabolites ratio** | | | | |
| NAA/Cr | 1.36 (1.058, 1.75) | 0.93 (0.576, 1.195) | -4.818 | <0.001 |
| Cho/Cr | 1.795 (1.433, 3.208) | 2.985 (1.79, 3.982) | -2.564 | 0.01 |
| Cho/NAA | 1.475 (0.996, 2.297) | 2.99 (2.09, 5.035) | -4.058 | <0.001 |
| **DTI histogram features** | | | | |
| FA_10Percentile | 0.072 (0.058, 0.088) | 0.076 (0.058, 0.093) | -1.138 | 0.255 |
| FA_90Percentile | 0.262 ± 0.056 | 0.307 ± 0.057 | -4.259 | <0.001 |
| FA_Energy | 270.711 (82.969, 1331.411) | 190.468 (95.888, 2497.332) | -0.558 | 0.577 |
| FA_Entropy | 0 (0, 0) | 0 (0, 0) | -1.865 | 0.062 |
| FA_InterquartileRange | 0.094 (0.073, 0.112) | 0.116 (0.097, 0.142) | -4.039 | <0.001 |
| FA_Kurtosis | 5.381 (4.146, 7.562) | 4.221 (3.495, 5.925) | -2.646 | 0.008 |
| FA_Maximum | 0.618 ± 0.166 | 0.704 ± 0.165 | -2.802 | 0.006 |
| FA_Mean | 0.153 (0.132, 0.18) | 0.181 (0.16, 0.2) | -3.895 | <0.001 |
| FA_MeanAbsoluteDeviation | 0.062 ± 0.016 | 0.072 ± 0.017 | -3.486 | <0.001 |
| FA_Median | 0.135 (0.112, 0.16) | 0.166 (0.145, 0.185) | -4.293 | <0.001 |
| FA_Minimum | 0.022 (0.016, 0.031) | 0.02 (0.015, 0.028) | -1.124 | 0.261 |
| FA_Range | 0.592 ± 0.176 | 0.681 ± 0.17 | -2.765 | 0.007 |
| FA_RobustMeanAbsoluteDeviation | 0.041 ± 0.013 | 0.05 ± 0.013 | -3.947 | <0.001 |
| FA_RootMeanSquared | 0.174 (0.15, 0.205) | 0.207 (0.183, 0.223) | -4.050 | <0.001 |
| FA_Skewness | 1.381 (0.964, 1.864) | 0.923 (0.686, 1.311) | -3.895 | <0.001 |
| FA_TotalEnergy | 350.876 (100.834, 1551.878) | 1261.881 (124.23, 3047.885) | -1.464 | 0.143 |
| FA_Uniformity | 1 (1, 1) | 1 (1, 1) | -1.320 | 0.187 |

| | | | | |
|---|---|---|---|---|
| FA_Variance | 0.008 (0.005, 0.011) | 0.01 (0.007, 0.016) | -2.586 | 0.010 |
| MD_10Percentile | 1042.686 ± 165.194 | 971.811 ± 169.221 | 2.280 | 0.024 |
| MD_90Percentile | 1741.727 (1537.543, 1970.405) | 1825.906 (1530.627, 2130.594) | -0.890 | 0.374 |
| MD_Energy | 25500760758.029 (6250367857.747, 114223541307.714) | 15457335828 (6374650537.833, 99821830238.82) | -0.171 | 0.864 |
| MD_Entropy | 5.282 ± 0.551 | 5.502 ± 0.525 | -2.201 | 0.030 |
| MD_InterquartileRange | 322.357 (250.221, 480.62) | 409.755 (296.447, 531.316) | -1.995 | 0.046 |
| MD_Kurtosis | 3.422 (2.691, 4.47) | 3.785 (2.692, 4.983) | -0.829 | 0.407 |
| MD_Maximum | 2631.202 (2161.414, 3032.256) | 3037.228 (2445.871, 3480.182) | -2.359 | 0.018 |
| MD_Mean | 1410.234 ± 207.235 | 1394.653 ± 300.788 | 0.323 | 0.748 |
| MD_MeanAbsoluteDeviation | 198.856 (150.652, 278.815) | 251.439 (206.14, 345.225) | -2.635 | 0.008 |
| MD_Median | 1337.95 (1240.557, 1537.193) | 1357.409 (1132.607, 1513.879) | -0.901 | 0.368 |
| MD_Minimum | 556.049 (281.594, 712.756) | 548.99 (346.94, 637.015) | -0.633 | 0.527 |
| MD_Range | 2200.009 ± 973.774 | 2546.465 ± 882.268 | -2.010 | 0.047 |
| MD_RobustMeanAbsoluteDeviation | 135.604 (103.85, 202.288) | 172.67 (132.662, 239.533) | -2.182 | 0.029 |
| MD_RootMeanSquared | 1390.62 (1286.763, 1584.484) | 1454.452 (1241.13, 1608.724) | -0.127 | 0.899 |
| MD_Skewness | 0.451 (-0.202, 0.662) | 0.725 (0.308, 1.249) | -3.193 | 0.001 |

| | | | | |
|---|---|---|---|---|
| MD_TotalEnergy | 29969071622.907 (6268349405.238, 137030570190.491) | 58516072087.423 (8565893235.055, 152838922350.748) | -0.823 | 0.410 |
| MD_Uniformity | 0.031 (0.025, 0.04) | 0.026 (0.021, 0.035) | -1.961 | 0.050 |
| MD_Variance | 66104.613 (40389.001, 123171.297) | 110100.638 (74677.166, 230287.738) | -2.807 | 0.005 |
| **DTI clinical parameters** | | | | |
| FA2d_Maximum | 0.158 (0.121, 0.221) | 0.256 (0.205, 0.309) | -4.961 | <0.001 |
| FA2d_Maximum_ratio | 0.302 (0.244, 0.406) | 0.465 (0.361, 0.66) | -4.619 | <0.001 |
| FA2d_Mean | 0.104 (0.084, 0.134) | 0.176 (0.143, 0.215) | -5.387 | <0.001 |
| FA2d_Mean_ratio | 0.266 (0.2, 0.361) | 0.423 (0.31, 0.538) | -4.398 | <0.001 |
| FA2d_Median | 0.099 (0.078, 0.125) | 0.166 (0.135, 0.204) | -5.160 | <0.001 |
| FA2d_Median_ratio | 0.263 (0.19, 0.337) | 0.434 (0.308, 0.507) | -4.337 | <0.001 |
| FA2d_Minimum | 0.059 (0.046, 0.075) | 0.096 (0.072, 0.141) | -4.793 | <0.001 |
| FA2d_Minimum_ratio | 0.244 (0.143, 0.411) | 0.384 (0.228, 0.517) | -2.553 | 0.011 |
| MD2d_Maximum | 1561.38 (1404.408, 1791.777) | 1365.195 (1130.26, 1598.15) | -3.475 | <0.001 |
| MD2d_Maximum_ratio | 1.683 (1.467, 2.041) | 1.555 (1.282, 1.88) | -2.105 | 0.035 |
| MD2d_Mean | 1407.612 ± 295.907 | 1129.315 ± 220.195 | 5.772 | <0.001 |
| MD2d_Mean_ratio | 1.732 ± 0.444 | 1.431 ± 0.322 | 4.209 | <0.001 |
| MD2d_Median | 1407.771 ± 316.357 | 1126.54 ± 231.221 | 5.492 | <0.001 |
| MD2d_Median_ratio | 1.74 ± 0.473 | 1.427 ± 0.337 | 4.080 | <0.001 |
| MD2d_Minimum | 1201.092 ± 284.493 | 916.584 ± 205.267 | 6.139 | <0.001 |
| MD2d_Minimum_ratio | 1.62 (1.364, 1.894) | 1.27 (1.112, 1.446) | -4.613 | <0.001 |

Note: IDH, isocitrate dehydrogenase; MRS, magnetic resonance spectroscopy; Cho, Choline; NAA, N-acetyl-aspartate; Cr, Creatine; DTI, diffusion tensor imaging; FA, fractional anisotropy; MD, mean diffusivity.

Supplementary Table S3. Logistic regression results for prediction IDH status based on features from mono-modal MRI model.

| Variable | | OR with CI | SE | Wald | *p* value |
|---|---|---|---|---|---|
| **Conventional MRI** | enhancement degree | 2.88 (1.54-5.40) | 0.321 | 10.912 | <0.001 |
| | necrosis/cyst | 4.63 (1.60-13.44) | 0.543 | 7.957 | 0.005 |
| **$^{1}$H-MRS** | Cho/NAA | 1.53 (1.16-2.01) | 0.14 | 9.315 | 0.002 |
| | NAA/Cr | 0.82(0.47-1.41) | 0.279 | 0.523 | 0.469 |
| **DTI histogram** | FA-Maximum | 2.08(0.98-4.41) | 0.383 | 3.652 | 0.056 |
| | FA-Median | 1.245(0.29-5.36) | 0.743 | 0.089 | 0.77 |
| | FA-RootMeanSquared | 1.05(0.25-4.34) | 0.725 | 0.004 | 0.95 |
| | FA-Skewness | 0.39(0.18-0.85) | 0.395 | 5.627 | 0.018 |
| | MD-Entropy | 1.72(1.03-2.87) | 0.261 | 4.296 | 0.038 |
| | MD-Skewness | 1.51(0.91-2.51) | 0.259 | 2.517 | 0.113 |
| **Conventional DTI** | $FA_{mean}$ | 2.82(1.61-4.96) | 0.288 | 12.972 | <0.001 |
| | $MD_{mean}$ | 0.83(0.30-2.36) | -0.182 | 0.118 | 0.731 |
| | $MD_{min}$ | 0.32(0.11-0.95) | -1.131 | 4.225 | 0.040 |

Supplementary Table S4. Features differences from mono-modal MRI between MGMT-methylated group and MGMT-unmethylated group.

| Variable | methylated, N = 37 | unmethylated, N = 17 | Z/t | *p* value |
|---|---|---|---|---|
| **Conventional MRI** | | | | |
| Sex, n (%) | | | 0.440 | 0.507 |
| Female | 16 (43.2%) | 9 (52.9%) | 1.229 | 0.222 |
| male | 21 (56.8%) | 8 (47.1%) | | |
| Age, Median (IQR) | 62(51, 68) | 52(48, 63) | -1.23 | 0.219 |
| Tumor location, n (%) | | | | 1 |
| Frontal lobe | 12(32.4%) | 6(35.3%) | | |
| Parietal lobe | 6(16.2%) | 2(11.8%) | | |
| Temporal lobe | 15(40.5%) | 7(41.2%) | | |
| Other | 4(10.8%) | 2(11.8%) | | |
| Enhancement degree, n (%) | | | | 0.547 |
| marked | 28 (75.7%) | 15 (88.2%) | | |
| mild | 7 (18.9%) | 1 (5.9%) | | |
| no | 2 (5.4%) | 1 (5.9%) | | |
| Necrosis or cyst, n (%) | | | | 0.696 |
| no | 5 (13.5%) | 3 (17.6%) | | |
| yes | 32 (86.5%) | 14 (82.4%) | | |
| Nonenhancing part, n (%) | | | 0.032 | 0.857 |
| no | 14 (37.8%) | 6 (35.2%) | | |
| yes | 23 (62.2%) | 11(64.7%) | | |
| Peritumoral edema, n (%) | | | | 0.915 |
| 0 | 4(10.8%) | 2(11.8%) | | |
| 1 | 20(54.1%) | 8(47.1%) | | |

| | | | | |
|---|---|---|---|---|
| 2 | 13(35.1%) | 7(41.2%) | | |
| **MRS metabolites ratio** | | | | |
| NAA/Cr | 1.03 (0.736, 1.43) | 0.72 (0.522, 0.98) | -2.468 | 0.014 |
| Cho/Cr | 3.14 (2.06, 4.73) | 2.2 (1.69, 3) | -1.891 | 0.060 |
| Cho/NAA | 2.85 (2.06, 5.11) | 3.14 (2.14, 4.9) | -0.074 | 0.941 |
| **DTI histogram features** | | | | |
| FA_10Percentile | 0.086 (0.072, 0.092) | 0.075 (0.057, 0.087) | -1.425 | 0.154 |
| FA_90Percentile | 0.315 ± 0.05 | 0.295 ± 0.05 | 1.319 | 0.193 |
| FA_Energy | 3814.293 (1547.159, 5444.654) | 1297.514 (199.005, 4566.673) | -1.872 | 0.061 |
| FA_Entropy | 0.001 (0, 0.006) | 0.002 (0, 3.359) | -0.849 | 0.396 |
| FA_InterquartileRange | 0.12 ± 0.026 | 0.117 ± 0.036 | 0.341 | 0.734 |
| FA_Kurtosis | 4.561 (3.719, 6.804) | 5.266 (4.485, 6.45) | -0.717 | 0.473 |
| FA_Maximum | 0.883 (0.752, 0.978) | 0.778 (0.709, 0.89) | -1.760 | 0.078 |
| FA_Mean | 0.186 (0.173, 0.206) | 0.179 (0.146, 0.191) | -1.946 | 0.052 |
| FA_MeanAbsoluteDeviation | 0.073 ± 0.013 | 0.072 ± 0.019 | 0.205 | 0.840 |
| FA_Median | 0.172 (0.16, 0.197) | 0.162 (0.125, 0.174) | -2.226 | 0.026 |
| FA_Minimum | 0.02 (0.015, 0.03) | 0.019 (0.015, 0.023) | -0.773 | 0.440 |
| FA_Range | 0.858 (0.753, 0.975) | 0.756 (0.7, 0.869) | -1.741 | 0.082 |
| FA_RobustMeanAbsoluteDeviation | 0.05 ± 0.01 | 0.049 ± 0.015 | 0.229 | 0.820 |
| FA_RootMeanSquared | 0.209 (0.195, 0.227) | 0.207 (0.179, 0.213) | -1.872 | 0.063 |
| FA_Skewness | 1.082 ± 0.48 | 1.239 ± 0.421 | -1.60 | 0.251 |
| FA_TotalEnergy | 3814.293 (2238.243, 5444.654) | 2968.95 (1297.514, 4566.673) | -1.574 | 0.116 |
| FA_Uniformity | 1 (0.999, 1) | 1 (0.131, 1) | -0.887 | 0.375 |

| | | | | |
|---|---|---|---|---|
| FA_Variance | 0.009 (0.007, 0.012) | 0.011 (0.009, 5.79) | -1.481 | 0.139 |
| MD_10Percentile | 977.89 ± 176.449 | 989.934 ± 146.312 | -0.245 | 0.807 |
| MD_90Percentile | 1887.749 ± 526.035 | 1906.492 ± 410.802 | -0.130 | 0.897 |
| MD_Energy | 10780356889.835 (7118404022, 94131997089.284) | 19568940063 (4459202779.942, 149328791522.544) | -0.473 | 0.473 |
| MD_Entropy | 5.477 ± 0.561 | 5.594 ± 0.457 | -0.755 | 0.453 |
| MD_InterquartileRange | 409.183 (297.716, 517.113) | 431.75 (369.75, 576.323) | -0.605 | 0.545 |
| MD_Kurtosis | 3.572 (2.745, 4.823) | 4.031 (2.682, 4.886) | -0.065 | 0.948 |
| MD_Maximum | 2991.131 ± 754.786 | 3186.573 ± 861.932 | -0.845 | 0.402 |
| MD_Mean | 1400.067 ± 323.148 | 1437.302 ± 249.188 | -0.420 | 0.676 |
| MD_MeanAbsoluteDeviation | 251.971 (195.345, 344.176) | 306.66 (229.395, 343.589) | -0.717 | 0.473 |
| MD_Median | 1361.172 (1112.34, 1528) | 1402.973 (1258.225, 1513.841) | -0.587 | 0.557 |
| MD_Minimum | 548 (408.363, 636.687) | 576.049 (307.219, 621.78) | -0.121 | 0.904 |
| MD_Range | 2497.961 ± 847.821 | 2698.183 ± 966.055 | -0.771 | 0.444 |
| MD_RobustMeanAbsoluteDeviation | 175.06 (132.047, 237.303) | 180.997 (151.845, 229.518) | -0.512 | 0.609 |
| MD_RootMeanSquared | 1450.806 ± 350.553 | 1492.71 ± 272.33 | -0.435 | 0.665 |
| MD_Skewness | 0.641 (0.311, 1.153) | 0.724 (0.239, 1.154) | -0.233 | 0.816 |
| MD_TotalEnergy | 18952517534.088 (7735794378.461, 107057308469.268) | 149328791522.544 (13914716667.495, 236881968852.389) | -1.946 | 0.052 |

| | | | | |
|---|---|---|---|---|
| MD_Uniformity | 0.027 (0.022, 0.036) | 0.023 (0.019, 0.032) | -0.996 | 0.319 |
| MD_Variance | 99733.282 (75521.389, 204087.381) | 138777.549 (81615.48, 233815.145) | -0.847 | 0.397 |
| **DTI clinical parameters** | | | | |
| FA2d_Maximum | 0.288 (0.193, 0.329) | 0.242 (0.205, 0.271) | -0.773 | 0.440 |
| FA2d_Maximum_ratio | 0.514 ± 0.202 | 0.474 ± 0.194 | 0.686 | 0.496 |
| FA2d_Mean | 0.177 ± 0.065 | 0.166 ± 0.048 | 0.635 | 0.528 |
| FA2d_Mean_ratio | 0.44 ± 0.159 | 0.411 ± 0.144 | 0.642 | 0.524 |
| FA2d_Median | 0.174 ± 0.068 | 0.16 ± 0.05 | 0.718 | 0.476 |
| FA2d_Median_ratio | 0.432 ± 0.169 | 0.404 ± 0.145 | 0.606 | 0.547 |
| FA2d_Minimum | 0.11 ± 0.053 | 0.097 ± 0.038 | 0.946 | 0.349 |
| FA2d_Minimum_ratio | 0.427 ± 0.22 | 0.351 ± 0.202 | 1.213 | 0.231 |
| MD2d_Maximum | 1375.498 ± 314.319 | 1446.83 ± 312.213 | -0.776 | 0.441 |
| MD2d_Maximum_ratio | 1.514 ± 0.401 | 1.664 ± 0.339 | -1.336 | 0.187 |
| MD2d_Mean | 1131.217 ± 234.915 | 1143.407 ± 197.863 | -0.186 | 0.853 |
| MD2d_Mean_ratio | 1.4 ± 0.33 | 1.451 ± 0.275 | -0.555 | 0.581 |
| MD2d_Median | 1123.928 ± 245.298 | 1141.254 ± 209.11 | -0.252 | 0.802 |
| MD2d_Median_ratio | 1.392 ± 0.344 | 1.45 ± 0.293 | -0.597 | 0.553 |
| MD2d_Minimum | 924.713 ± 215.834 | 922.592 ± 204.69 | 0.034 | 0.973 |
| MD2d_Minimum_ratio | 1.289 ± 0.311 | 1.299 ± 0.357 | -0.100 | 0.921 |

Note: MGMT, O6-methylguanine-DNA methyltransferase; MRS, magnetic resonance spectroscopy; Cho, Choline; NAA, N-acetyl-aspartate; Cr, Creatine; DTI, diffusion tensor imaging; FA, fractional anisotropy; MD, mean diffusivity.

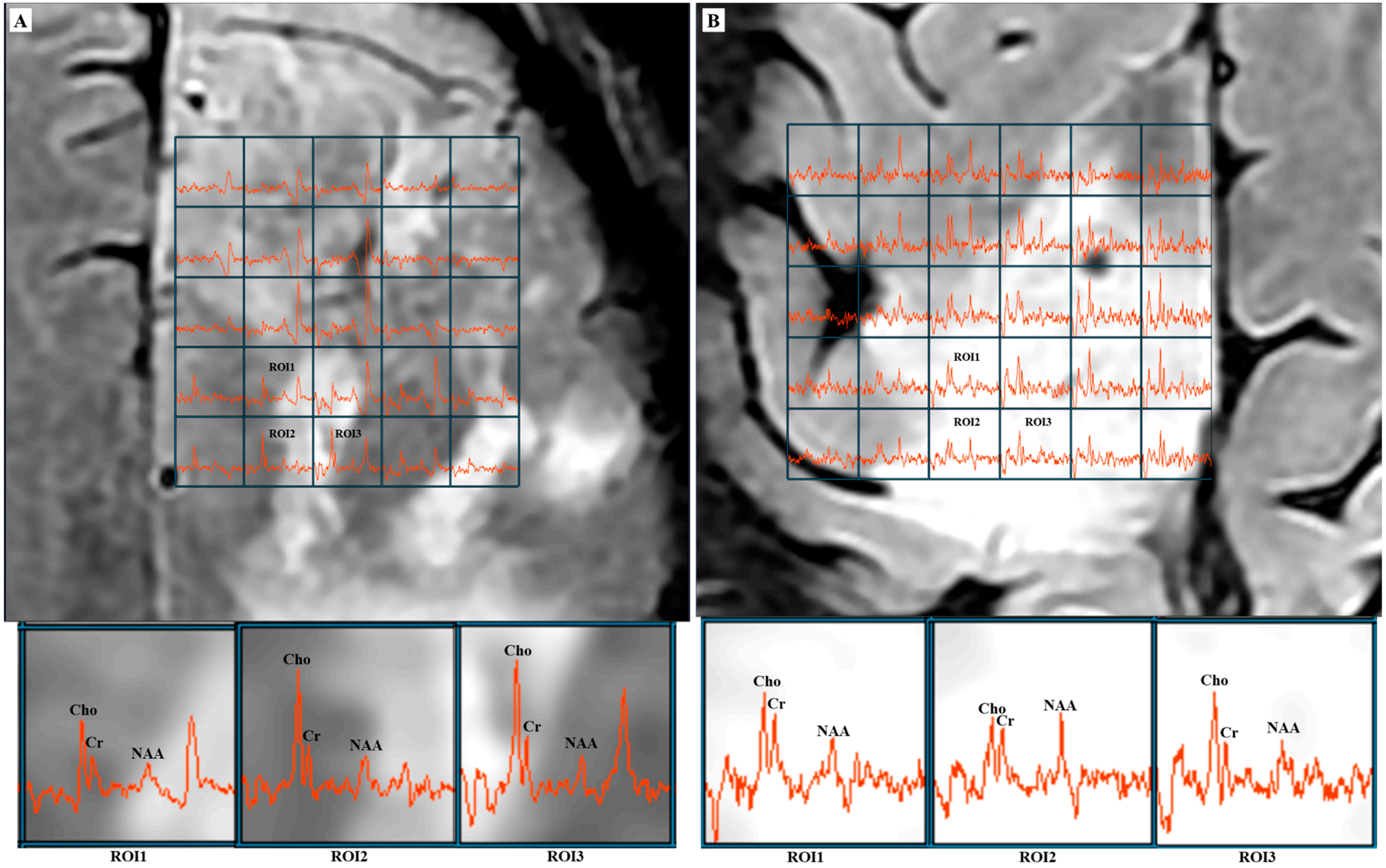


Supplementary Figure S1. The ROI selection in multi-voxel 2D MRSI spectra for cases in the IDH-wildtype group and IDH-mutant group. (**A**) ROI selection in a case with

IDH-widltype glioblastoma. (**B**) ROI selection in a case with IDH-mutant astrocytoma.

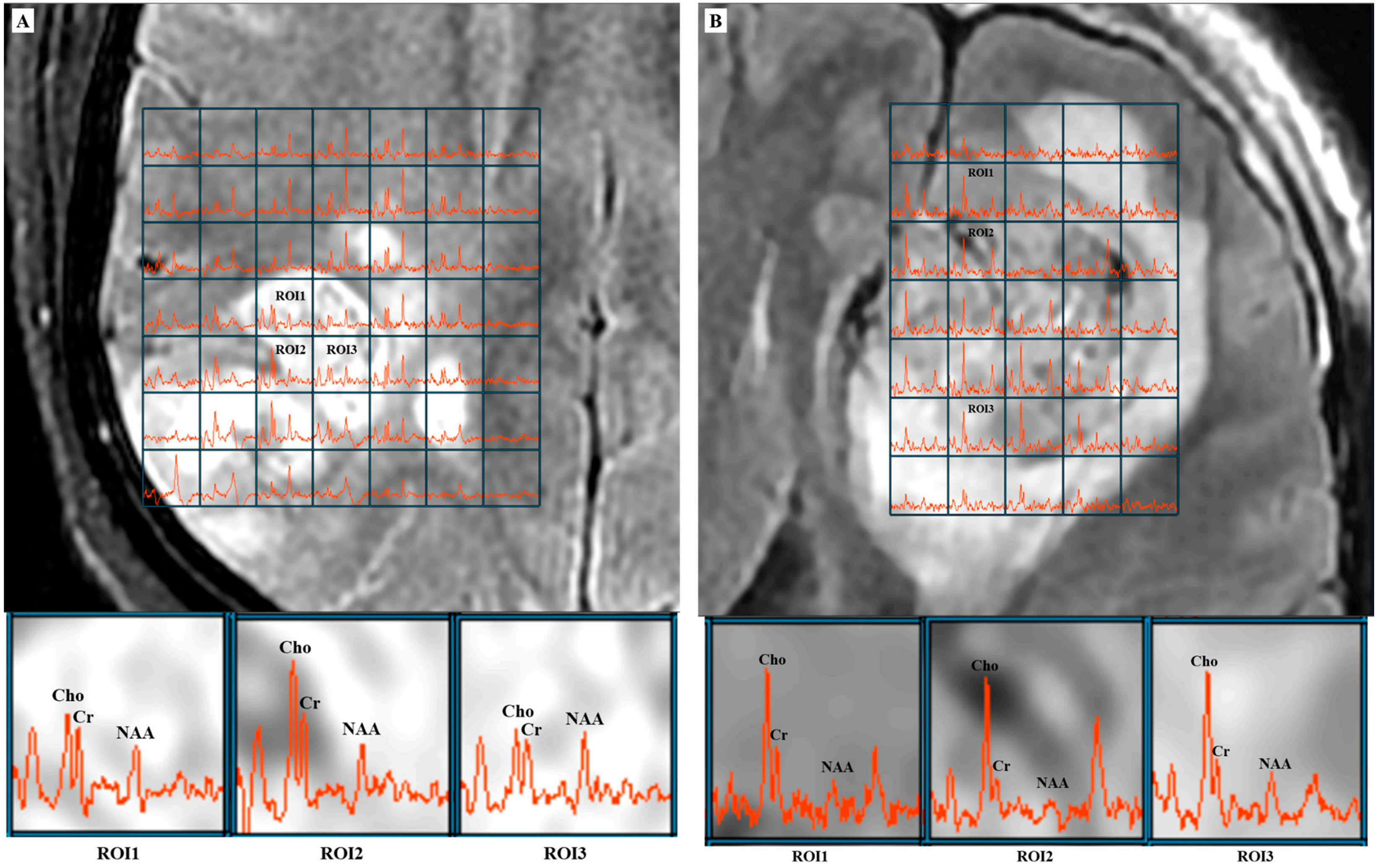

Supplementary Figure S2. The ROI selection in multi-voxel 2D MRSI spectra for cases in the MGMT-methylated group and MGMT-unmethylated group. (**A**) ROI selection in a glioblastoma case with methylated-MGMT promoter. (**B**) ROI selection in a glioblastoma case with unmethylated-MGMT promoter.
